# Factors Influencing the Transborder Transmission of Brucellosis in Cattle Between Côte d'Ivoire and Mali: Evidence From Literature and Current Key Stakeholders

**DOI:** 10.3389/fvets.2021.630580

**Published:** 2021-03-10

**Authors:** Wilfried Délé Oyetola, Kanny Diallo, Katharina Kreppel, Philippe Soumahoro Kone, Esther Schelling, Bassirou Bonfoh, Rianatou Bada Alambedji

**Affiliations:** ^1^Ecole Inter-Etats des Sciences et Médecine Vétérinaires, Dakar, Senegal; ^2^Centre Suisse de Recherches Scientifiques en Côte d'Ivoire, Abidjan, Côte d'Ivoire; ^3^Nelson Mandela African Institution of Science and Technology, Arusha, Tanzania; ^4^Emergency Center for Transboundary Animal Diseases, Food and Agriculture Organization of the United Nations, Kinshasa, Democratic Republic of Congo; ^5^Vétérinaires Sans Frontières Suisse, Bern, Switzerland

**Keywords:** brucellosis, livestock mobility, transborder surveillance, Côte d'Ivoire, Mali

## Abstract

Brucellosis is one of the main zoonoses affecting ruminants. Cattle and small ruminants are involved in transhumance and trade between Côte d'Ivoire and Mali. The endemic nature of the disease in both countries, connected through transhumance, poses unique challenges and requires more information to facilitate disease surveillance and the development of integrated control strategies. This study aimed to assess the main factors influencing the historical and current transborder transmission of brucellosis between Côte d'Ivoire and Mali. A literature review was conducted and data collection was performed through a participatory, transdisciplinary process by holding focus group discussions and interviews with key stakeholders. Cattle breeders, herdsmen, professionals of animal and human health, border control agents and experts took part. The data was analyzed to generate essential new knowledge for transborder brucellosis transmission factors and control strategies. From the literature, the seroprevalence of brucellosis in both countries varied from 11% (1987) to 20% (2013) and 15% (1972–1973) to 5% (2012–2014) in Mali and Côte d'Ivoire, respectively. The reduction of seroprevalence in Côte d'Ivoire was the result of the annual vaccination campaigns which lowered it from 28% (1978) to 14% (1984) after an increase due to livestock policy implemented in 1976. The meta-analysis and interviews jointly showed that the cross-border mobility was associated with the livestock development policy in Côte d'Ivoire as well as the ECOWAS act on the free movement of people and goods. This act supported the seasonal transhumance of livestock for access to pasture land in southern humid zones in Côte d'Ivoire. The seasonal mobility for grazing and trade was the main risk factor for the spread of brucellosis between pastoral zones of both countries. The existing legal health framework and border control mechanism do not achieve transborder surveillance to control brucellosis. Existing sanitary regulations should be adapted at regional scale to integrate a joint surveillance of high priority zoonotic diseases like brucellosis at border controls.

## Introduction

Brucellosis is a neglected zoonotic disease widely present in Sub-Saharan Africa (1) where the most common bacterial species are *Brucella abortus* and *Brucella melitensis* (2). It has been declared a priority zoonosis in Côte d'Ivoire (3) and Mali (4), and the only strain identified in the area is *B. abortus* (5–7), affecting cattle, small ruminants and humans.

The economic losses due to brucellosis in animals are due to a decline in fertility in cows, resulting from abortions, and the reduction of milk production. In Mali, decreased fertility and lower milk production have been estimated to be 20 and 16%, respectively (8). In Côte d'Ivoire, it was estimated that sedentary breeders lose 10% of their annual income to brucellosis related causes (9). The disease is also a public health concern with human infections in rural Côte d'Ivoire (5.3%) (10) and urban Mali (7.7%) (11). In humans, the infection is often confused with other febrile illnesses, resulting in ineffective treatment, high treatment costs and morbidity resulting in the inability to work (12, 13).

In animals, the main symptom suggesting brucellosis is abortion; however, following chronic infection, swelling of the testes and arthritis (hygroma) can also be observed (14, 15). Clinical diagnosis is not easy and makes border checks difficult as infection must be confirmed by laboratory diagnosis.

The transmission of *Brucella* occurs through direct contact and through environmental contamination. The bacteria are in biological secretions of infected animals such as sexual secretions, milk, aerosols and abortive materials (15). Animal infection occurs during communal herding and grazing, the addition of infected animals to a herd, transhumance movement and mixing at livestock markets. All these situations arise in the context of cross-border mobility. Therefore, the risk of contagious disease spread between countries with seasonal transhumance and cross-border cattle trade is high (16).

In West Africa, the cross-border mobility of livestock is guaranteed by the free movement of people and goods between member countries of the Economic Community of West African States (ECOWAS) with the International Transhumance Certificate as one of the control tools (17, 18). This allows Sahelian pastoralists (Mali) to access the livestock markets and pastoral resources in coastal countries (Côte d'Ivoire). Cattle from Mali constitute 60% of the cattle marketed in Côte d'Ivoire (19) which, like other coastal countries, secure their food and access to affordable meat this way and also supply their livestock. However, as a consequence, cross-border zoonotic disease spread is likely to present a serious problem as seen in other areas of Africa (20).

Cattle brucellosis seroprevalence in Mali (19.7%) (21) and the north of Côte d'Ivoire (4.6%) (10) constitutes a threat to public health and a burden to the rural economy. *Brucella* is persisting in both countries albeit with different seroprevalences, after Côte d'Ivoire introduced a control program, but failed to eradicate the disease. New approaches for brucellosis control considering transborder livestock mobility are essential to reduce the impact of the disease on public health and the economy. For this, it is important to understand the factors which can explain the involvement of livestock mobility in brucellosis transmission.

Using metadata from existing literature and databases, and carrying out a qualitative investigation into the present situation can provide important insights. The objective of this study was to describe the cross-border risk factors of transmission that would explain the seroprevalence dynamics of brucellosis in cattle in Côte d'Ivoire and Mali.

## Materials and Methods

### Study Area

Both parts of this study were carried out for Côte d'Ivoire and Mali, two bordering countries and members of ECOWAS. Mali is a landlocked Sahelian country with one of the largest cattle populations in the West African sub-region with an estimated 10,622,750 cattle, 16% of which is in the Sikasso region bordering north Côte d'Ivoire (22). Côte d'Ivoire is a coastal country situated in a humid climate zone with 85% of cattle located in the north (23). In addition to the continuous flow of live animals imported from Mali to supply the Ivorian market, each year, according to local administrative authorities at the border of Tengrela and Odienne, transhumance for grazing is legally practiced in the north of Côte d'Ivoire between November to April by mobile pastoralists from Mali.

**Figure 1:**
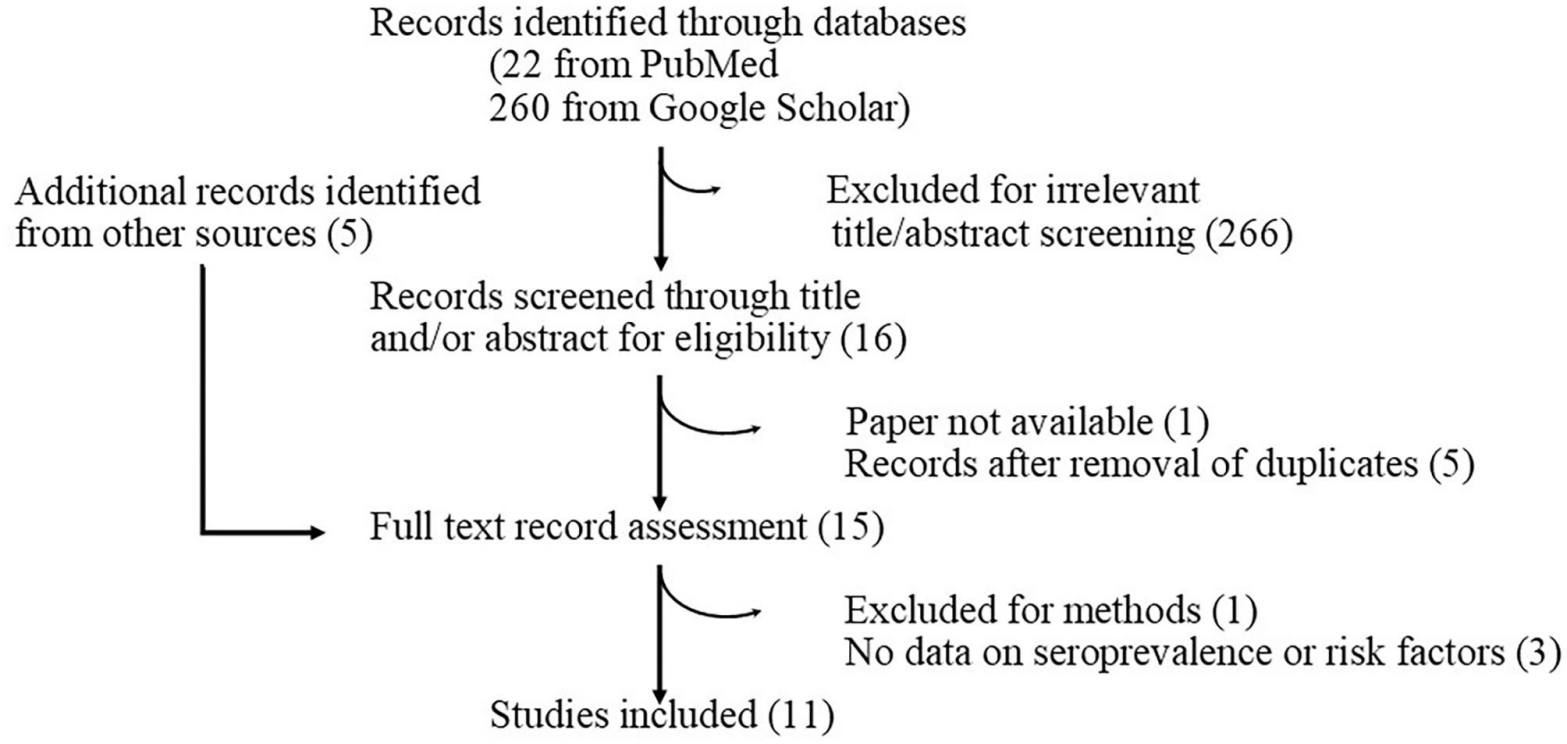
Flow diagram summarizing process of literature search.

Field investigations were conducted in seven high risks departments in north and west Côte d'Ivoire: Korhogo, Niakaramandougou (Niakara), Ferkessedougou (Ferké), Tengrela, Boundiali, Odienne and Man as well as at the border inspection post of Zegoua located in the circle of Kadiolo in the south of Mali (Figure 1). Each one of these sites has a livestock market supplied by cattle from cross-border trade. Except for the department of Man, all other localities are transit or reception areas for cross-border transhumance. The border inspection post of Zegoua is a veterinary control post located at the border area of the main axis of cross-border trade of livestock between Mali and Côte d'Ivoire (24).

### Literature Review for Situational Analysis on Brucellosis

A systematic literature review and meta-analysis for the following outcomes were conducted: (1) seroprevalence of brucellosis in Mali and/or Côte d'Ivoire cattle; (2) transhumance between Mali and Côte d'Ivoire; (3) factors related to brucellosis in cattle in Mali and/or Côte d'Ivoire; and (4) intervention and policy regarding brucellosis in cattle in Mali and/or Côte d'Ivoire. Google Scholar and Pubmed were used to identify relevant articles irrespective of publication date using the following terms: brucellosis OR brucella AND Côte d'Ivoire OR Mali OR “French Sudan.”

**Figure 2:**
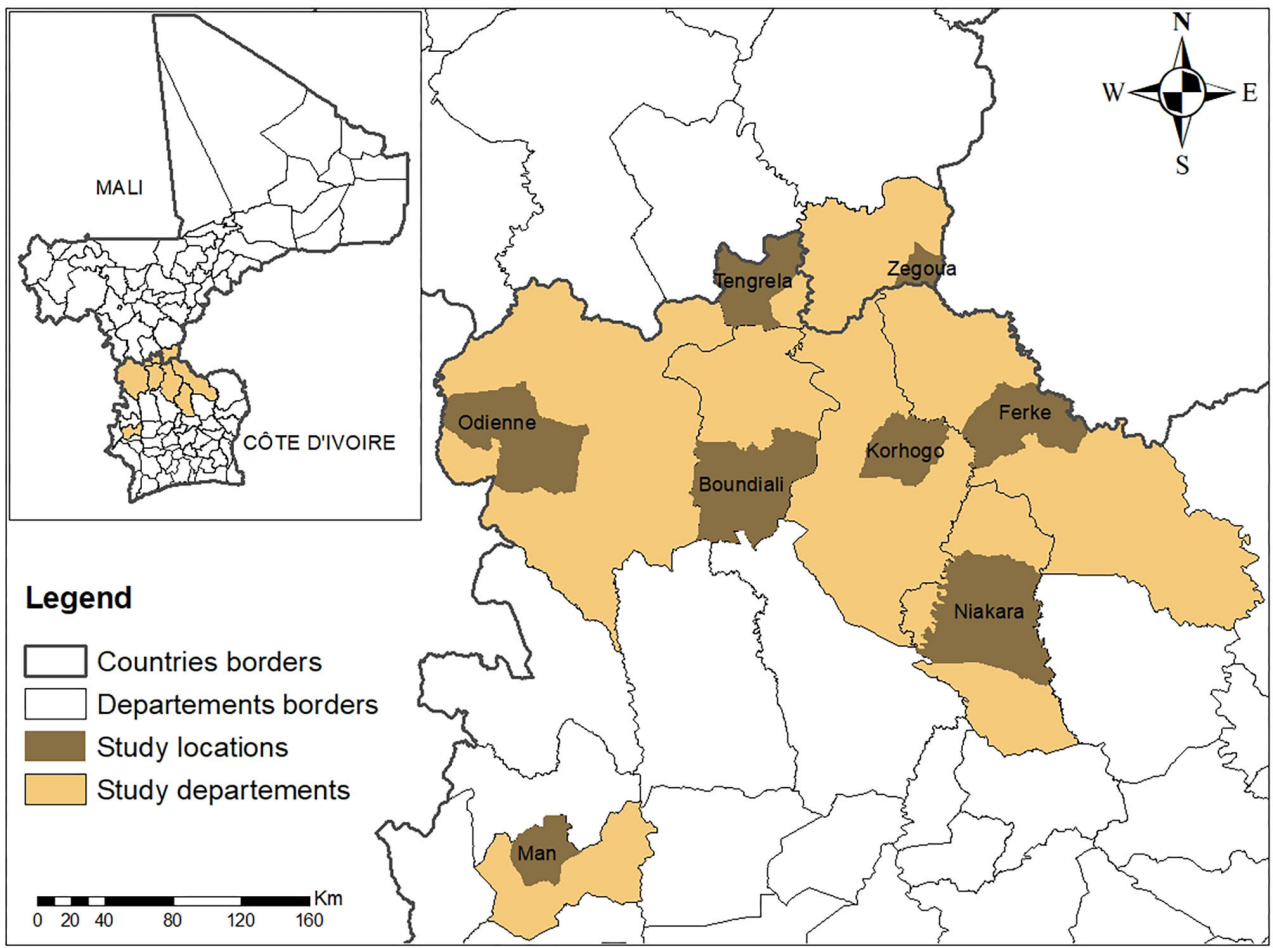
Locations of departments in Côte d'Ivoire and the border office in Mali.

The database findings were systematically screened according to the title and/or abstract in English and French. Only relevant papers were collected and reviewed (Figure 2). A paper was deemed relevant if the title or the abstract suggested that the study was related to brucellosis, contained an estimation of infection rate or risk factors linked to infection, and was carried out in Côte d'Ivoire and/or Mali. Review studies were excluded but their bibliography was used to find additional sources. Target animal populations were cattle. Data extracted were: year of study, study zone, sample size, diagnostic tests used, number of positives, seroprevalence, and risk factors.

Due to the low number of available literature, the results of unpublished works, which were cited in the included studies, were taken into account when cattle seroprevalence results and year of the study were available. It was not possible to ascertain if the data discussed in studies come from pastoralist or sedentary herds.

Overall, with this method 11 publications were identified, from which 33 epidemiological studies on brucellosis seroprevalence were obtained (Table 1), 14 of which came from gray literature. They presented the epidemiological situation between 1972 and 2014 in Côte d'Ivoire and Mali. The data did not cover all time periods and missing values were observed for seroprevalence, between 1985 and 2003, and then between 2006 and 2012, for Côte d'Ivoire; prior to 1987 and between 1993 and 2006, for Mali.

**Table 1:** Seroprevalence of brucellosis in cattle in Côte d'Ivoire and Mali between 1972 and 2014.

**Study number**	**Study year**	**Country**	**Zone**	**Serological test**	**Cattle sampled**	**Cattle positive**	**Prevalence (95% CI)**	**References**
1	1972–1973	Côte d'Ivoire	North, West, Southwest	SAW, CFT	779	116	14.9	(25)
2	1975–1977	Côte d'Ivoire	All the country	SAW, RBT, CFT	12,343	1,341	10.8	(26)
3	1976	Côte d'Ivoire	North, Center, South	SAW, RBT	5,552	557	10	(5)*
4	1977	Côte d'Ivoire	North, Center, South	SAW, RBT	5,322	688	12.9	(5)*
5	1978	Côte d'Ivoire	North, Center, South	SAW, RBT	5,987	657	11	(5)*
6	1978	Côte d'Ivoire	North	SAW, RBT	1,180	334	28.3	(9)
7	1979	Côte d'Ivoire	North, Center, South	SAW, RBT	4,305	901	20.9	(5)*
8	1980	Côte d'Ivoire	North, Center, South	SAW, RBT	3,472	566	16.3	(5)*
9	1981	Côte d'Ivoire	North, Center, South	SAW, RBT	3,509	547	15.6	(5)*
10	1982	Côte d'Ivoire	North, Center, South	SAW, RBT	6,485	815	12.6	(5)*
11	1983	Côte d'Ivoire	North, Center, South	SAW, RBT	4,643	580	12.5	(5)*
12	1984	Côte d'Ivoire	North, Center, South	SAW, RBT	1,956	282	14.4	(5)*
13	1987	Mali	Livestock areas	–	8,276		11.4	(6)
14	1989–1990	Mali	District of Bamako	–			19.7	(6)
15	1989–1990	Mali	Livestock area of CIPEA	–			22.4	(6)
16	1989–1990	Mali	Niono circle	–			32.1	(6)
17	1989–1990	Mali	Yanfolila circle	–			21.4	(6)
18	1991	Mali	All the country excepted Gao and Bamako	ELISA	1,000		23.3	(6)
19	1992	Mali	–	–			26.4	(27)
20	1994	Mali	District of Bamako	–			23.3	(27)
21	1998	Mali	All the country	RBT			8.7	(27)
22	2001	Mali	All the country	RBT			19.5	(27)
23	2004	Côte d'Ivoire	South (Traditional farm)	SAW, RBT, ELISA, CFT	137		4.3 (1.3–8.7)	(28)
	2004	Côte d'Ivoire	South (Dairy farm)	SAW, RBT, ELISA, CFT	244		3.6 (1.1–7.1)	(28)
24	2005	Côte d'Ivoire	Center	SAW, RBT, ELISA, CFT	611		8.8 (5–16.4)	(29)
25	2007	Mali	All the country	–	801	112	13.98	(21)
26	2008	Mali	All the country	–	268	37	13.8	(21)
27	2009	Mali	All the country	–	483	25	5.17	(21)
28	2010	Mali	All the country	–	552	64	11.59	(21)
29	2009–2010	Mali	Cinzana	RBT	204	2	1.0	(30)
30	2011	Mali	All the country	–	723	55	7.6	(21)
31	2012	Mali	All the country	–	1072	123	11.47	(21)
32	2013	Mali	All the country	–	809	160	19.77	(21)
33	2012–2014	Côte d'ivoire	North	RBT, ELISA	473		4.6 (2–10.6)	(10)

*SAW, Slow agglutination of Wright; RBT, Rose Bengal Test; CFT, Complement Fixation Test; ELISA, Enzyme-Linked Immunosorbent Assay; CIPEA, Centre International pour l'Elevage en Afrique (International center for livestock in Africa); CI, Confidence interval*.

* *Author's calculation based on data from the source*.

Online databases of the Food and Agricultural Organization of the United Nations (FAOSTAT) were explored to access complementary data about cross-border animal trade of living cattle in Côte d'Ivoire and Mali. Type of mobile transaction (importation or exportation), number of herds, origin of importation and destination of exportation were searched in FAOSTAT (31). This database considers only data from trade by truck and train, and obtained from official data, FAO estimate including those made with trading partners.

There is no existing online database on transhumance. Reports of organizations or institutions working on cross-border transhumance between Mali and Côte d'Ivoire were also searched.

R software (version 4.0.2) was used to test the relationship between the import trade flow and the brucellosis seroprevalence in Côte d'Ivoire using the Shapiro-Wilk normality test and the Pearson correlation coefficient (32). Only the years (1972–1973, 1976–1984, 2004, 2005, and 2012–2014) for which seroprevalence data was available were used. In cases where two studies were conducted in the same year, the one with the larger study population was selected. For studies carried out over several years, the average annual number of animals imported over this period was calculated.

### Cross-Border Data Collection

Data was collected at the border from four different sources: (1) veterinary health professionals; (2) public health professionals; (3) breeders and herdsmen; and (4) animal health experts. The knowledge, attitude and perception of stakeholders were assessed and experts' opinion was recorded.

A multidisciplinary team composed of a veterinarian, a social scientist and a biologist conducted discussions based on semi-structured questionnaires in French and in the local language about knowledge and practices on brucellosis diagnostic and control strategies from March to April 2018 with livestock actors and professionals of public and veterinary health.

Target professionals from veterinary health services were representatives of the public sector or the laboratory in each locality and private practitioners from the veterinary clinic of the locality. From the veterinary health sector, 2 veterinarians from the veterinary laboratory, 9 veterinarians and livestock engineers from public veterinary services including border control, and 7 private veterinarians and veterinary technicians from across study locations in Côte d'Ivoire, as well as the head of animal border control office in Mali were interviewed individually. They provided information on: capacities to perform brucellosis diagnosis in animals including in border control and control activities for brucellosis.

Target professionals from public health services were the representatives of the public sector and any other actors suggested by the representatives. From the public health sector, except from Man, Ferké and Zegoua, individual interviews were recorded with 6 physicians, 2 nurses, 1 midwife, 1 laboratory assistants and 2 technicians in charge of reporting health statistics. Data collected included their knowledge of brucellosis, their institution's capacity to perform brucellosis diagnosis and involvement in the control of brucellosis.

Qualitative data from 53 breeders and herdsmen was collected through focus group discussions (FGD) in Niakara, Ferké, Tengrela, Boundiali, and Odienne, based on an invitation from the head of the local veterinary services. In addition, 12 breeders and herdsmen were interviewed individually with the same questionnaire used for focus group discussions during the visit of their herds in Korhogo, Niakara, Boundiali, and Odienne. Data collected from all livestock actors included the local name of brucellosis, known symptoms, possibility of care for infected animals, and perception of brucellosis control in the cross-border areas.

Between July and August 2020, a questionnaire was administered to 13 animal health experts by email and virtual interviews. The participants worked in the fields of epidemiology, microbiology, infectious disease pathology and sociology and were identified because of their track record (publications) on brucellosis in West and East Africa, mainly in Côte d'Ivoire and Mali, their professional activities in the field or laboratory, and their position in institutions engaged in research and control of zoonoses. Their opinion on factors explaining the spread and endemicity of brucellosis, reasons for brucellosis transmission risk from cross-border mobility, brucellosis detection and control strategy in sub-Saharan Africa focusing in Mali and Côte d'Ivoire, were collected.

These data were used to produce an Ishikawa diagram and conduct a Strengths, Weaknesses, Opportunities, and Threats (SWOT) analysis. The possible factors stated by the experts were listed and grouped into categories to construct the Ishikawa diagram and establish a cause-effect relationship of the reasons for the spread or endemicity of brucellosis in cross-border areas in both countries. A weight of importance (low, medium or high) was assigned to each category according to the number of points it accumulated. One point was one quotation of a factor by an expert. Importance of a category was estimated as being low, medium or high if it had a total of <10 points, between 10 and 20 points, and more than 20 points, respectively. A SWOT analysis of current control measures of brucellosis in both countries was extracted from the received responses.

## Results

### Metadata on Brucellosis Transmission Patterns

#### Environmental Factors in Transborder Brucellosis Spread

The results suggest that an increase in the frequency of droughts and the resulting reduction in grazing areas in Mali drove an increase in transhumance toward humid zones (Côte d'Ivoire). Evidence from the literature shows that in Mali, where the presence of brucellosis was suspected in 1930s in cattle (33, 34), livestock migration toward the southern coastal countries has increased from 1968, because of droughts (35) that reduced grazing areas. The first waves of migration of the Fulani zebu herds from southern Mali to northern Côte d'Ivoire took place in 1969 (36). The following year brucellosis were diagnosed in animal, which could explain the calf mortalities observed in livestock in Côte d'Ivoire (37). By the 1970s, ~15,000 cattle from Mali came via transhumance to the north of Côte d'Ivoire, each year (38).

In Mali, brucellosis was reported during several deficit rainfall periods, which resulted in major droughts and increased cross-border transhumance due to scarcity of grazing areas (35). In 1987, bovine seroprevalence was evaluated for the first time to be 11.4% (6). At the end of the 1980s, the seroprevalence rose to 22%. In the 1990s, the seroprevalence in Mali was higher than 20% with strong variations depending on locality. From 2000 to 2013, the seroprevalence estimates were always under 20% (Table 1).

#### Economic and Policy Factors in Transborder Brucellosis Spread

The study reveals an increase of brucellosis prevalence in Côte d'Ivoire from 1970 to 1978 due to livestock trade and policy. Seroprevalence and import numbers of live cattle were associated. The import of live cattle from various countries to Côte d'Ivoire decreased from 280,000 head in 1972 to 145,000 head in 1976 (31). The brucellosis seroprevalence also dropped from 14.9% (25) to 10% (5) during the same time. Imports increased and reached 175,000 head in 1978. The meta-data analysis of data from 1972 to 2014 showed a significant correlation (*p*-value < 0.001) between the seroprevalence and the level of cattle importation to Côte d'Ivoire (*r* = 0.88) explaining the role that cattle trade could play in the transborder transmission of brucellosis between both countries.

In 1976 the Ivorian government decided to settle pastoral Fulani from Mali and Burkina Faso, with the project ≪ Opération Zébu ≫ (39). This sedentary policy promoted the introduction of Zebu cattle from the Sahel to Côte d'Ivoire without any control of their serological status at the border. New outbreaks of brucellosis appeared as a result of the distribution of uncontrolled breeding nuclei. The brucellosis seroprevalence in cattle has thus increased, as illustrated in 1978, to 28.3% of cows in northern Côte d'Ivoire (9).

#### Brucellosis Control Strategies and Risk Factors From Literature

The results from our findings highlighted the efforts of Côte d'Ivoire to control brucellosis in cattle and the risk factors identified by scientists.

In Côte d'Ivoire, the “Société pour le Développement des Productions Animales” (SODEPRA), the national institution in charge of animal production development initiated annual brucellosis vaccination campaigns from 1978 to 1987 which aimed to reduce the incidence of abortions observed in sedentary cattle herds. An attenuated vaccine with strain B19 and an inactivated vaccine with strain H38 were used (40). The vaccination program reduced abortion and stillbirth rates by 37% after the first year of implementation (40) during which 34% of cows were vaccinated in northern Côte d'Ivoire (5). The seroprevalence studies in animals during each annual vaccination campaign revealed that the cross-border zone in the northern of Côte d'Ivoire, was the most infected region of the country (5). Following these control program, the seroprevalence had reduced to 14.4% in 1984 (5). Another vaccination program targeting local herds was implemented in Côte d'Ivoire, from 1990 until the shutdown of SODEPRA in 1994 (40, 41) without published data. However, seroprevalence results from 2004 published later, showed an additional drop. Indeed, the seroprevalence observed in Côte d'Ivoire in 2004, 2005 and between 2012 and 2014 were 4% in the south (28), 8.8% in the center (29) and 4.6% in the north (10), respectively, without significant differences linked to the region. The risk factors of cattle infection in Côte d'Ivoire reported in the literature were: animal age, herd size, grazing with small ruminants and contact with pastoralist herds (10, 42).

In Mali, none of the literature recorded in this article indicated that the implementation of brucellosis vaccination campaigns or stamping out programs. However, in 1997, the Malian government issued a decree providing guidance on the guidelines to be applied to an infected animal or herd. In addition, it gave the possibility to impose the vaccination of a herd containing infected cattle (43). The constraints of this law may have explained a drop in requests for brucellosis diagnosis by livestock owners, with the corollary of a relatively low seroprevalence 8.7% (27) observed in 1998.

### Qualitative Investigations of the Brucellosis Situation at the Border

#### Brucellosis Control and Assessment From Interviews

Currently, according to professionals of veterinary health interviewed (14/18) in this study, raising awareness about the existence of the disease and the risk of transmission through consumption of raw milk is the only action taken against brucellosis since the shutdown of SODEPRA in Côte d'Ivoire. Professionals of public health were not engaged in this informal public engagement led by the veterinarians. The situation was similar in Mali according to experts.

The SWOT analysis of current control measures of brucellosis in the cross-border area in both countries produced with responses of the 13 experts is shown in Table 2. The strengths of brucellosis control are perceived to be its prioritization in the frame of the Global Health Security Agenda using the One Health approach in both countries, and involvement of researchers, animal health professionals and some Non-Governmental Organizations in public engagement. The most relevant weaknesses of brucellosis control mentioned were the lack of a control strategy and funding allocation as well as limitations of laboratory capacities in brucellosis diagnostics. Experts argued that veterinary laboratories were not able to identify *Brucella* species or produce a vaccine because of their low biosafety level. Implementation of a One Health approach by using public health services especially laboratories to address the limited coverage of veterinary laboratories in some border localities was an opportunity identified in a SWOT analysis from experts' responses.

**Table 2:** SWOT analysis of brucellosis control in Côte d'Ivoire and Mali.

**Strengths**	**Weaknesses**
Disease classified as a priority zoonosis Interest and awareness by some stakeholders Presence of veterinary services in livestock areas Veterinary laboratories equipped for serological diagnosis Experience in the implementation of vaccination plan in Côte d'Ivoire	No national control strategy for brucellosis No collaboration between medical and veterinary services Funding system for control activities Limitations of laboratories capacities Diagnostic of brucellosis is just clinical Involvement of breeders
**Opportunities**	**Threats**
Framework provided by implementation of One health approach Existence of human health services laboratories, particularly those of the Institute Pasteur Availability of vaccines at international level	Reliability and completeness of current epidemiological data in humans and animals Interest/relevance of the fight for the breeders Security situation Mobility/trade

#### Detection Capacity of Brucellosis at the Border of Côte d'Ivoire and Mali

Representatives of veterinary control services at border points in both countries revealed that transborder surveillance is specific to the national regulations of each country. To facilitate pastoralists' cross-border transhumance following the free movement of goods and persons in the ECOWAS area, a legal framework has been adopted and established the International Certificate of Transhumance (ICT) in 1998. Although it should harmonize and ease cross-border mobility by providing herd traceability and assurance to the host country that animals are healthy and up to date with their regulatory vaccine(s), the ICT was seldom used. Instead, the health certificate issued by post in the area of departure, as well as the herd vaccination certificates were presented and accepted. The Brucella status of the herd was not mentioned in any of these certificates. Control at the border posts consists of checking of these documents and the visual inspection of animals without any laboratory diagnostics. According to one of the representatives interviewed “*it is easier to control cross-border mobility for animal trade compared to transhumance, because pastoralists did not always use the official transhumance routes*.”

According to all the professionals of veterinary health and experts interviewed, the laboratory diagnosis of brucellosis is officially performed by the Central veterinary laboratory (LCV) located at Bamako in Mali and the national agricultural development support laboratory (LANADA) which have a regional veterinary laboratory at Korhogo, in Côte d'Ivoire. These tests are performed in the frame of research activities. LANADA of Korhogo provided laboratories services for the northern of Côte d'Ivoire. The veterinary laboratories in both countries are equipped to perform mainly serological diagnosis of brucellosis (Rose Bengal Test, RBT). Neither performs serological surveillance at the borders of their respective countries. Experts from both countries raised the issue that molecular tools (Polymerase Chain Reaction, PCR) currently used in these laboratories for other purposes in their virology services could also be used for improve detection of brucellosis.

All professionals of public health interviewed in the study area mentioned that the hospital in each locality has laboratories. Although they do not currently perform brucellosis diagnostics, they could, if equipped with reagents to diagnose suspected samples of livestock and humans.

#### Stakeholder Knowledge, Attitude, and Perception

Among the professionals of public health interviewed 4 out of 12 remembered having heard of brucellosis during their academic training, but admitted that they were not able to explain the clinical features of the disease in humans. Only one professional described it as zoonosis. Veterinary practitioners admitted that they based their diagnosis on clinical signs: hygroma, unexplained abortion, orchitis, placental retention.

The presence of brucellosis was confirmed by pastoralists who participated in the FGDs in the cross-border area in northern of Côte d'Ivoire. They noted that it had become rarer and animal infection was not linked to the cross-border transhumance herds unlike some others diseases such as foot and mouth disease. They did not know the cause of brucellosis but were able to describe it. In Niakara and Tengrela, they described brucellosis as a painless swelling of knee or hock joint of animals. While, in Korhogo, Ferké, Boundiali and Odienne, they noted abortion as another possible sign.

Despite informal public engagement led by veterinarians, pastoralist showed limited knowledge and risky attitude toward brucellosis. The majority of participants in the locality of Ferké expressed that they do not agree with the veterinarian about the contagiousness of the disease. For example, one participant mentioned that “*the veterinarians say the disease is contagious, but when one animal is sick, the others don't get sick*.” During FGDs in Ferké, Niakara, Tengrela, Fulani herdsmen mentioned the ancestral benefits of raw milk consumption for the cow and for the Fulani themselves. For them, drinking raw milk is considered to give strength and boiled milk reduce the cow production according to their beliefs from tradition and their own observations. Raw milk is believed to be healthier, have therapeutic properties and contain more nutrients. For example, a breeder said that “*the ancestral habit of consuming raw milk is still very much ingrained among Fulani herdsmen who use milk production to supplement their income*.” In addition, Fulani Livestock farmers interviewed in the cross-border area in Côte d'Ivoire, claimed that animal brucellosis was curable thanks to a traditional treatment based on Fulani knowledge. This treatment involves incising the animal's hygroma. One participant in Odienne locality mentioned that “*to achieve complete healing, oxytetracycline (an antibiotic) must be administered after the incision*.”

#### Risk Factors for Brucellosis Transmission Between Côte d'Ivoire and Mali

**Figure 3:**
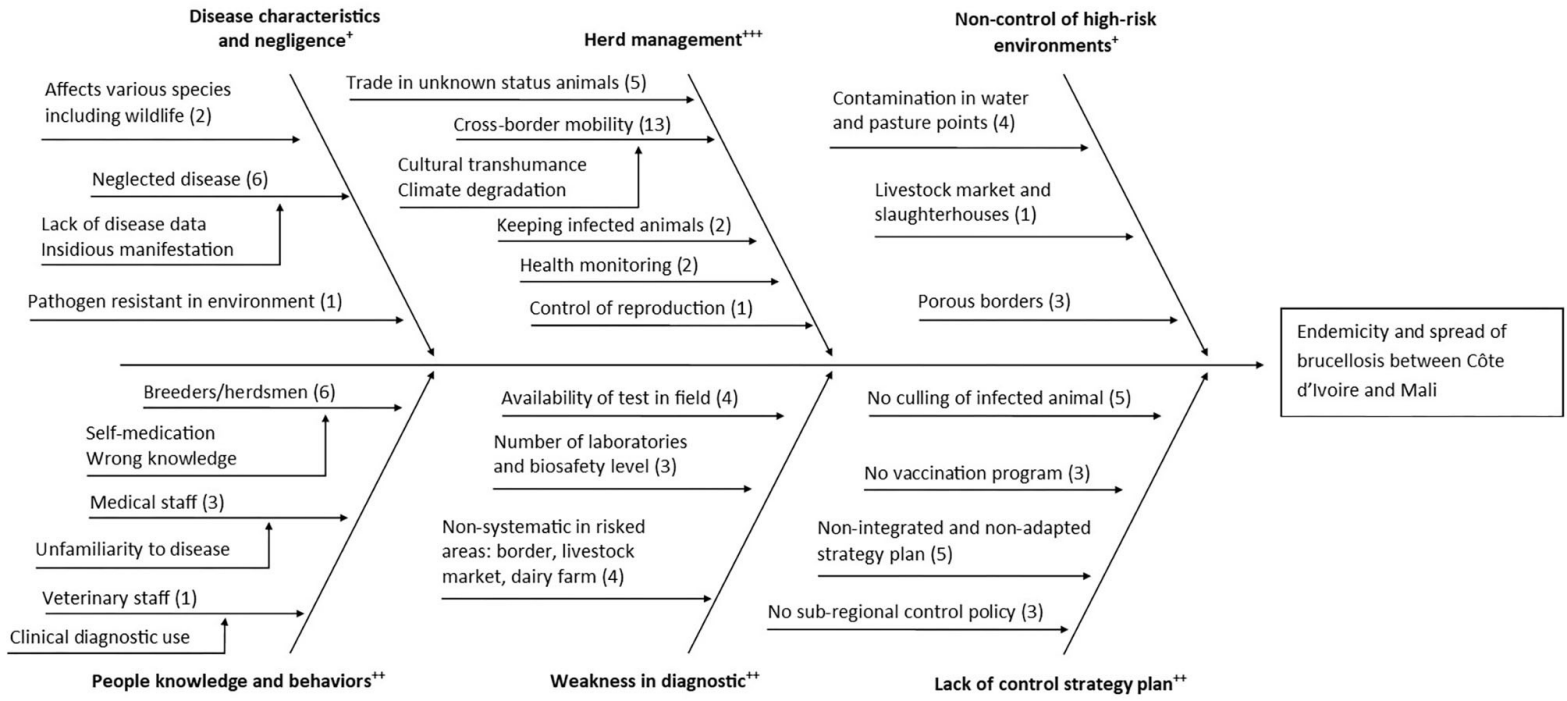
An Ishikawa diagram of causes of brucellosis persistence in Mali and Côte d'Ivoire. This diagram presents classification of causes identified by the experts that could lead to the endemicity and spread of brucellosis in sub-Saharan Africa mainly between Côte d'Ivoire and Mali. In parenthesis the number of experts who given the factor. The weight of each category: high (+ + +) medium (++) low (+).

Based on meta-analysis of data in the literature, the main factors for the spread of brucellosis in both countries identified were livestock policy and trading of live animals from uncontrolled transhumance or trade systems (livestock markets). Responses by experts have identified 21 factors for brucellosis endemicity between both countries and particularly in the cross-border area (Figure 3). The main factors according to their quotation frequency were: cross-border mobility (13/13), neglect of brucellosis (6/13), wrong knowledge and self-medication of breeders and herdsmen (6/13), no culling of infected animals (5/13) and the lack of integrated and adapted national strategic plans for control (5/13). Experts also explained the role of cross border mobility in transmission by other factors. The mentioned lack of brucellosis control measures in the countries of origin and no screening prior to crossing the borders of each country of either, animals from cross-border transhumance or trade. During transhumance, cattle are in contact with wildlife and local herds, but one of the experts considered the risk of Brucella transmission through this route as low, due to the short duration of these interactions most of the time. However, commercial cattle transactions, whether from transhumant or trade herds, lead to introduction of animals of unknown status into the local herds, making it a critical point of brucellosis spread within the markets network. All the factors described by the experts were classified into six categories with various weights. The high importance category was the causes related to uncontrolled herds management, which included the factors of cross-border mobility and trade in animals of unknown status in livestock markets. The medium importance categories were the causes related to lack of control strategy and policy, the causes related to weakness in diagnostic, and the causes related to wrong knowledge and practice of stakeholder. The low importance categories were the causes related to brucellosis characteristics and negligence, and the causes related to non-control of high-risk environments like water points, grazing, livestock markets where *Brucella* bacteria could survive thanks to their resistance or infection of other host like small ruminants or wildlife.

## Discussion

The objective of this study was to describe the cross-border risk factors of transmission to shed light on the seroprevalence dynamics of brucellosis in cattle in Côte d'Ivoire and Mali using metadata from literature and a current qualitative investigation. Main results from literature from 1970 to 2015, were the role of droughts and livestock policies that have led to an increase in cross-border transhumance, which is the main risk factor for brucellosis infection in cattle. Also, the study found a strong correlation between seroprevalence and cross border cattle trade; and the reduction of brucellosis seroprevalence in Côte d'Ivoire, probably thanks to a vaccination program. Results of interviews with professionals from veterinary and public health sectors showed redundancy of the International Certificate of Transhumance (ICT) to control brucellosis and lack of serological surveillance at border level. FGDs revealed limited knowledge and risky attitude of breeders and herdsmen. According to expert interviews, in addition to cross-border mobility, the causes related to uncontrolled herd management, lack of control strategy and policy as well as brucellosis characteristics and neglect, explained endemicity and spread of brucellosis in both countries.

The results of this study revealed that livestock cross-border mobility promoted the endemicity and spread of brucellosis between Côte d'Ivoire and Mali. This cross-border mobility is the effect of the seasonal transhumance which allows herds to access natural resources and trade to supply the Ivorian market. Both types of mobility were also at the core of the reintroduction of brucellosis to the Czech Republic from neighboring countries in 1973 and 1974, respectively, despite the protective measures taken by the country (44).

Transhumance is one of the most widely practiced traditional pastoral systems with flexibility and adaptability to environmental constraints, exacerbated by droughts. Droughts in the Sahel in the 1980s led to significant losses of Mali's cattle (45). The renewal of livestock from the end of 1980s and in 1990s could explain the increase of brucellosis prevalence during this period in Mali before a declined. The nature of transhumance is to provide flexible and continuous grazing and water to cattle throughout the year, which is likely to become more important with increasing effects of climate change (46, 47). Transhumance promotes mixing between herds on grazing land and at water points. Contact between herds is a risk factor for disease transmission for both sedentary and transhumant herds (48). Contact with pastoralist herds was identified as a risk factors for cattle infection in the north of Côte d'Ivoire and seropositivity increased on average by 20% for each additional year of life (10), probably because of additional seasonal interaction with animals in transhumance. The fact that the north of Côte d'Ivoire, where cross-border transhumance was hosted and which benefited from a pastoralists sedentarization policy (39), was the most infected area of the country (5) until implementation of vaccination, suggested that livestock development policy and transhumance played a strong role in the spread of brucellosis.

Cross-border trade supplied not only slaughterhouses, but also Ivorian livestock markets, from which livestock farmers also obtain their supplies. Up to 5% of cattle from transhumant herds are sold in the livestock markets during seasonal transhumance (49). The risk of disease spread from livestock cross-border markets is around 80% if the disease seroprevalence is 1% and basic reproduction number around 1.25 (16). This means that supplying the Ivorian market with cattle from Mali, where brucellosis seroprevalence was 20% (21) would represent a very high risk of the spread of the disease in Côte d'Ivoire and may explain endemicity.

In the view of the processes by which transhumance and cross-border trade promote transmission, such as the need for contact and promiscuity, one might think that transhumance could be more implicated in brucellosis transmission than trade. Indeed, during transhumance contamination could be made by close contact with other herds, indirect transmission via contaminated environment, and the sale of animals to new herds. For trade, mainly the last point is of importance.

The results from of the implementation of a vaccination campaign in Côte d'Ivoire have shown that it is a strong strategic tool to reduce the impact of brucellosis in a risk area (40). In consequence, disruption of brucellosis vaccination in 1994 (40, 41), would be a factor promoting the maintenance of brucella spread. The local production of vaccine although not necessarily a priority, could support the practice of vaccination as control tool at national level. However, production would have to be accompanied by willingness and necessity by law to reach good vaccination coverage. In addition, vaccination alone is not enough to control or even eradicate brucellosis (50). Other measures, such as systematic testing and elimination of infected animals when prevalence is low, should be considered in addition to efficient surveillance.

The legal harmonization of transborder surveillance between ECOWAS countries represents a significant step forward in the control of transboundary diseases. However, the ICT used for sanitary control and traceability of herds needs to be revised to take into account the concerns of border countries on priority zoonoses and its use needs to be popularized among pastoral communities. A brucellosis free herd certification program in Mali and Côte d'Ivoire, could provide a monitoring tool for both transhumant and commercial herds. Capacity building of border control posts would involve regular laboratory diagnostic to improve transborder surveillance. The capacity of laboratories to perform Rose Bengal Test is a positive point for brucellosis screening. However, the use of PCR would allow in addition to the diagnosis of disease to characterize isolates from field for better epidemiological knowledge on strains (51).

Brucellosis is relatively unknown by breeders and herdsmen. They did not consider abortion was not mentioned as a sign of brucellosis in all the locations; this difference could be explained by their limited knowledge of the disease and association of abortion to other, more frequent diseases. Assimilation of hygroma and infection (14) is not enough to achieve an early detection of brucellosis in the cross-border area, as this symptom only appears in chronically infected animals. Other symptoms like abortion and orchitis could be used for community surveillance. Breeders behaviors regarding the consumption of raw milk and the incision of hygromas could favor the contamination of consumers and operators but also that of the environment. While *Brucella* does not survive long in a dry and sunny environment, it can still lead to the infection of other herds sharing the same space during the same day; this period of environmental contamination is longer during wet conditions and with presence of organic matter (15). For example, animals from a herd in transhumance in a contaminated pasture could be infected and spread the disease to other localities. The risk of transmission of brucellosis to humans due to some practices supported the need for a One Health approach in the management of infectious cases in animals. Thus, the detection of an animal outbreak could serve as an alert to the potential for human infection and vice versa. In addition, the possibility that laboratories of public health in locations remote from veterinary laboratories may be able to perform diagnostic tests should be exploited as part of the One Health approach. Surveillance of the causes of acute febrile illness (AFI) in humans could also be an indicator for monitoring brucellosis. Indeed, in East Africa it is estimated that brucellosis represented between 2.6 to 22.4% of AFI (13).

The disease has insidious and asymptomatic to non-specific manifestations which have led to its underestimation, neglect, and difficulty in raising financial support for investments in solutions or control programs compared to high mortality diseases. This could explain the lack of a control strategy plan including culling of infected animals which would be compensated for, or a vaccination program. However, although neither the economic impact in the cross-border area in the both countries nor that on public health are well known due to the insidious nature of the disease, the cumulative net losses between 2005 and 2015 due to brucellosis have been estimated at XOF 14.5 billion (95% CI: 6,278 × 10^6^-22,906 × 10^6^) or USD 23.6 million in cattle in Cote d'Ivoire (52).

According to the hierarchy of the categories of factors obtained from the Ishikawa diagram, it can be concluded that the fight against brucellosis should be achieved by improving herd management through the purchase of animals recognized as infection free, the control of reproduction with free animals, the quarantine and elimination of infected individuals, and the practice of cross-border mobility of livestock in compliance with the control measures of the countries in the zone. Also, countries with livestock mobility linkages should establish harmonized strategic plans that specify the standard operating procedures for brucellosis cases (culling of infected animals, vaccination of herds, etc.). They must also implement active surveillance at borders, livestock markets and dairy farms support by laboratories with improved diagnostic capabilities. All of this must be supported by awareness campaigns to increase knowledge of medical staff and pastoral communities about brucellosis.

Limitations of this study were the lack of data on transhumance and limited publications such as on seroprevalence and impact of vaccination program probably due to difficulties of the monitoring and evaluation of the programme and lack of digital storage access and loss of SODEPRA archives. To address the lack of data particularly from Mali, gray literature from the national livestock services of Mali, being relayed in published articles, was accepted. In many African countries, historically, published scientific literature is scarce. The contribution of sedentary and transhumant herds to the spread of brucellosis could not be explored because there was not enough published data specific to each population; however, the importance of mobility suggested that transhumant herds would be spreaders of the infection. Another obstacle leading to limitations of this study were administrative constraints, preventing the investigation of the public health sector in Man, Ferké and Zegoua. With regard to expert opinions available for this study, there may be bias as most of them are from the veterinary field. For future studies, inclusion of data on seroprevalence from small ruminants and humans may give a better view of the situation but this was beyond the scope of this study. Indeed, first human cases are caused by *B. melitensis* in Mali (33, 34) although this specie was not identify in animals in west Africa (7) and naturally infecting small ruminants (15) which are also involved in livestock mobility. Future studies should therefore consider including small ruminants. Better access to internal reports of some research or governmental institutions is needed to gain deeper insight into the health issues related to transhumance. While solutions to reduce disease spread such as moving of cattle by truck and livestock movement corridors may work in other settings, the socio-political implications make this a challenge in a cross-border context (53). Transhumance corridors exist, but cannot be solely dedicated to cross-border transhumance, moreover, they do not prevent identified contacts with sedentary herds, such as contamination of the environment or the acquisition of probably infected animals. It is also questionable if restricting movement will not simply increase contact between herds and drive Brucellosis prevalence. The reason behind cross-border transhumance is not to sell cattle but primarily to successfully raise livestock throughout the year. Mobility provides continuous access to grazing and water resources for livestock at low cost, which would be difficult to achieve by moving them by truck. However, during transhumance around 5% of cattle in transhumance are sell to resolve some problem such as culture damage (49) and the risk from this contamination way must be consider.

In conclusion, we found evidence that the evolution of brucellosis seroprevalence rates and through it, the circulation of brucellosis between Côte d'Ivoire and Mali, is influenced by cross-border mobility through seasonal transhumance and cross-border trade due to inadequate tools used by cross-border surveillance. It would be appropriate to re-evaluate the system and tools of cross-border surveillance for the control of brucellosis.

The cost-benefit ratio and the possible effectiveness of different control strategies adapted to the African context have to be assessed such as brucellosis vaccination, systematic control tests during border inspection or in market, infected-elimination and non-*Brucella* herd certification to control transmission of this cross-border disease. The first intervention would be the revision of the list of diseases monitored in the International Certificate of Transhumance to introduce brucellosis. These interventions will be discussed with stakeholders, based on the results obtained in a research project on surveillance-response in a cross-border context of which this study is a part.

## Data Availability Statement

The datasets generated and analyzed during the present study are available from the corresponding author upon request.

## Ethics Statement

The studies involving human participants were reviewed and approved by “Comité d'Ethique de la Recherche de l'Université Cheikh Anta Diop de Dakar - Sénégal” and “Comité National d'Ethique des Sciences de la Vie et de la Santé (CNESVS) du Ministère de la Santé et de l'Hygiène Publique of Côte d'Ivoire.” The ethics committee waived the requirement of written informed consent for participation. The animal study was reviewed and approved by “Comité d'Ethique de la Recherche de l'Université Cheikh Anta Diop de Dakar - Sénégal” and “Comité National d'Ethique des Sciences de la Vie et de la Santé (CNESVS) du Ministère de la Santé et de l'Hygiène Publique of Côte d'Ivoire.”

## Author Contributions

WO, ES, PK, BB, and RB conceived and designed the study. WO, RB, and BB coordinated collection of expert opinions. WO and RB coordinated field activities. WO conducted the literature search, field activities and collection of expert opinions, carried out the analysis, and wrote a first draft of the manuscript. WO, KD, KK, ES, BB, and RB read and approved the submitted manuscript. All authors contributed to the manuscript revision.

## Conflict of Interest

The authors declare that the research was conducted in the absence of any commercial or financial relationships that could be construed as a potential conflict of interest.
