# Supplementary material for: Factors Influencing the Transborder Transmission of Brucellosis in Cattle Between Côte d'Ivoire and Mali: Evidence From Literature and Current Key Stakeholders
Source: Front Vet Sci. 2021 Mar 10;8:630580. doi: 10.3389/fvets.2021.630580 (PMC7987678; doi:10.3389/fvets.2021.630580)
Supplement: Supplementary file 1 [file Data_Sheet_1.PDF]

FICHE DE RECUEIL D'AVIS D'EXPERT
Expert Opinion Collection Form

Dans le cadre de la rédaction d'un article qui vise à expliquer le maintien de la brucellose en Côte d'Ivoire et au Mali en utilisant à la fois une revue de la littérature et une approche participative, nous souhaiterions obtenir votre avis d'expert.
In the context of writing an article which aims to explain the maintenance of brucellosis in Côte d'Ivoire and Mali using both literature review and participatory approach, we would like to seek your expert opinion.

(i) Quels sont selon vous les principaux facteurs pouvant expliquer le maintien de la brucellose en Afrique subsaharienne ?
What do you consider to be the main factors that may explain the maintenance of brucellosis in sub-Saharan Africa?

(ii) La mobilité transfrontalière représente t'elle un risque pour le maintien de la brucellose ? Pourriez-vous argumentez votre réponse.
Is cross-border mobility a risk for the maintenance of brucellosis? Please, could you explain your answer.

(iii) Quel est votre opinion sur la détection de la brucellose en Afrique de l'Ouest (en Côte d'Ivoire et au Mali particulièrement) ?
What is your opinion on the detection of brucellosis in West Africa (especially in Côte d'Ivoire and Mali)?

(iv) Quels ont été les points forts et les points faibles de la lutte contre la brucellose en Côte d'Ivoire et au Mali ?

What have been the strengths and weaknesses of brucellosis control in Côte d'Ivoire and Mali?

**Points forts** ***(Strengths)***	**Points faibles** ***(Weaknesses)***

(v) Selon votre expertise, quels sont les points forts et les points faibles, les opportunités et les menaces de la surveillance transfrontalière pour le contrôle de la Brucellose entre les pays frontaliers (cas du Mali et de la Côte d'Ivoire) ?
According to your expertise, what are the strengths and weaknesses, opportunities and threats of cross-border surveillance for the control of Brucellosis between border countries (case of Mali and Côte d'Ivoire)?

**Forces *(Strengths)***	**Faiblesses *(Weaknesses)***

Opportunités (Opportunities)	***Menaces (Threats)***
