# Supplementary material for: Factors Influencing the Transborder Transmission of Brucellosis in Cattle Between Côte d'Ivoire and Mali: Evidence From Literature and Current Key Stakeholders
Source: Front Vet Sci. 2021 Mar 10;8:630580. doi: 10.3389/fvets.2021.630580 (PMC7987678; doi:10.3389/fvets.2021.630580)
Supplement: Supplementary file 2 [file Data_Sheet_2.PDF]

Guide for Focus Group Discussion

**General informations**	
Date:<br>Department:<br>Number of participants:	
**Questionnaire**	**Additional questions**
What are the common diseases in your department?	in animals?<br>in humans (your herdsmen and their families, your family and yourself)?
Do you know or have you ever heard of brucellosis?	Can you describe the clinical signs in animals? What is the local name of the disease?
Is brucellosis present in your department?	If so, how often?<br>Are there any unexplained abortions in your community?<br>Are there any cattle with swollen knees in your community?
Can brucellosis be transmitted to humans?	How can humans become infected?<br>Do you consume raw milk in your community?<br>Do you consume bovine blood in your community?<br>Do you assist cows during calving? If so, how?
What is the cause of brucellosis?	Is it related to herds from other countries? Or to your animal movements?
Is brucellosis curable?	If so, can you tell us who treats it? What treatment is used?
What do you think about the control and fight against brucellosis in your locality and at the borders?	Are there any actions against brucellosis?

Questionnaire for professionals from veterinary sector

**Identification of the respondent**	
Name:<br>Qualification/private or public sector:<br>Location (department):	
**Questionnaire**	**Additional questions**
What animal diseases are prevalent in your area?	
Does brucellosis exist in your area?	If so, how common is it?
How is brucellosis diagnosed in your department?	
What are the means of brucellosis control in your department and at the borders?	
How is border inspection carried out in your area?	Are samples taken for the laboratory?
Do you collaborate with other state services in your department?	With medical services? On what occasions? Border control services?

Questionnaire for professionals from medical sector

**Identification of the respondent**	
Name:<br>Qualification:<br>Location (department):	
**Questionnaire**	**Additional questions**
What are the human illnesses that are prevalent in your medical area?	Which zoonotic disease
Do you know or have you ever heard of brucellosis?	Can you define or describe it in humans?
Did you have any human case of brucellosis in your medical area?	
Does your institution carry out brucellosis diagnosis?	Have you already carried out the diagnosis of brucellosis in your institution?<br>Do you have laboratory capacities to diagnose brucellosis in humans or animals?
Does your institution intervene in brucellosis control?	If so, how?<br>Do you collaborate with the veterinary services?
